# Supplementary material for: Nowcasting the COVID‐19 pandemic in Bavaria
Source: Biom J. 2020 Dec 1;63(3):490–502. doi: 10.1002/bimj.202000112 (PMC7753318; doi:10.1002/bimj.202000112)
Supplement: Supplementary file 2 — Supporting Information [file BIMJ-63-490-s001.zip › code_and_data/code_public/2_evaluation/supp_note_1.pdf]

Nowcasting the COVID-19 pandemic in Bavaria

Supplemental Note: Evaluation of the nowcasting approach

Felix Günther, Andreas Bender, Katharina Katz, Helmut Küchenhoff, Michael Höhle

Introduction

We perform an evaluation of the Bayesian hierarchical nowcasting based on synthetic data mimicking the reported Bavarian COVID–19 data and retrospectively on the official data from the LGL that was reported until July, 31.

Synthetic data

Data generating process

For simulation of the synthetic data, we utilized a smoothed version of the observed number of reported disease onsets per day and specified a reporting delay model similar to the model described in equation 3 of the manuscript. In this discrete time hazard model for the reporting delay, we specified a linear time effect on the log-hazard with five change points over time. The aggregated number of newly diseased cases (green, solid) and number of reported cases per day (red, dotted) are:

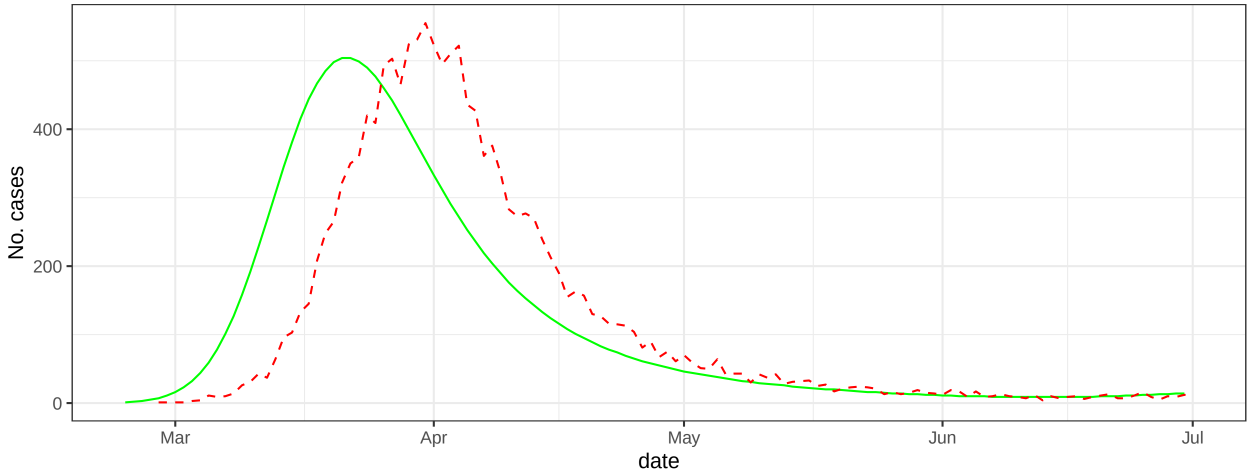

Figure 1: Daily numbers of newly diseased cases and reported cases in synthetic data

The changepoints in the linear time effect of the delay distribution lead to (smooth) changes in the delay distribution. We did not add any further effects of e.g., weekdays to the delay distribution model, and simulated reporting dates for each case with disease onset on day t directly from the delay distribution without adding any further variability.

The utilized delay distribution can be illustrated by plotting the empirical median and 25%- and 75%-quantile of the sampled reportig delay over time:

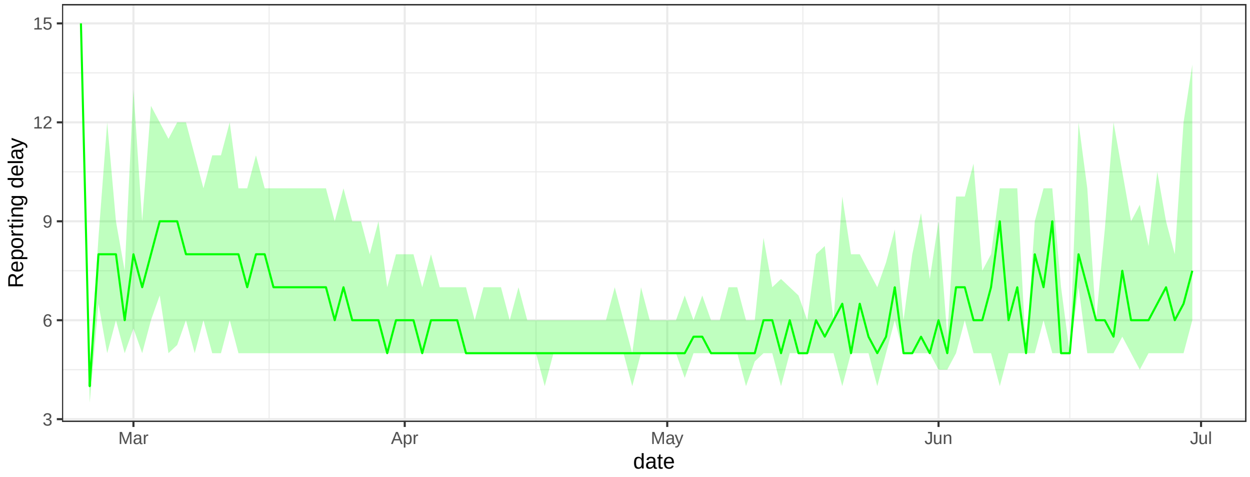

Figure 2: Empirical reporting delay of the synthetic data over time

Results

We esimated nowcasts for all dates t from March, 17 (22 days after disease onset of first case) until June, 30 by restricting the data to all cases reported until the respective date and compared the nowcast predicitions for all days $t-6,\dots,t-2$ to the actual true numbers of newly diseased cases per day.

Nowcasts are performed based on six different models (see description in the manuscript), and the performance of the models is compared via proper scoring rules, the root mean squared error and coverage frequencies of 95% prediction intervals.

In the following table, we present results based on the nowcasts for all dates and restricted to the time period between March, 17 and April, 30, where most of the dynamic in the epidemic curve happened:

Table 1: Quantification of the performance of six different nowcast models on synthetic data. Shown are the average metrics over all nowcast dates (*_f) and restricted to the period until May, 1 (*_m). Reported scores are the continuous ranked probability score, logarithmic score, root mean squared error of posterior median, and coverage frequencies of 95% prediction intervals. Estimated models are Poisson and Negative Binomial models with 1) (assumed) constant delay distribution, 2) linear time-effects with changepoints every two weeks before now (*_cp2W), 3) daily changes in delay distribution based on a first-order random walk prior (*_rW) and 4) true changepoints of the data generating process (*_cptrue).

model	crps_f	crps_m	logS_f	logS_m	rmse_f	rmse_m	cov_f	cov_m
poisson_rW_const	46.68	88.93	13.24	23.29	89.75	127.58	0.53	0.20
poisson_rW_cp2W	12.53	24.77	3.68	4.86	36.22	53.11	0.95	0.90
negBinom_rW_cp2W	12.47	24.61	3.68	4.86	36.01	52.78	0.95	0.90
negBinom_rW_rW	28.37	59.41	3.90	5.43	92.33	135.77	0.91	0.81
poisson_rW_cptrue	11.88	23.40	3.63	4.77	35.31	51.76	0.95	0.91
negBinom_rW_cptrue	11.90	23.47	3.62	4.78	35.48	52.01	0.96	0.92

In the following plot we show nowcast predictions and 95%-PIs 3 days before the current date over time for each model, and compare them with the true number of newly diseased cases:

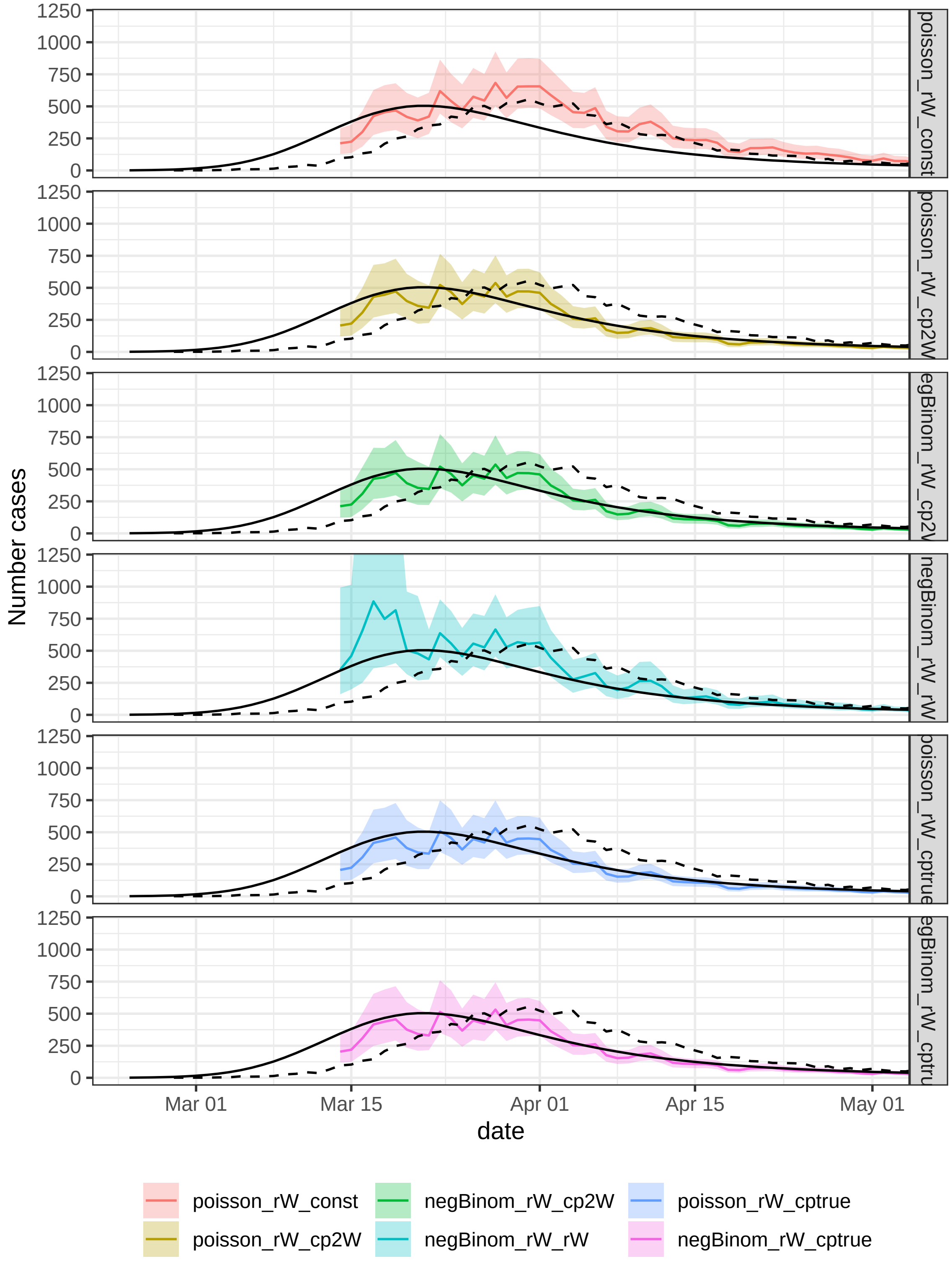

Figure 3: Nowcast predictions and 95% PIs for nowcasts 3 days before 'now' compared to true number of disease onsets (solid) and number of reported cases (dotted).

Note that in the quantitative evaluation based on scores and coverage frequencies, we focus on nowcasts 2-6 days before now and not only 3 days before.

We can also compare the estimated delay distribution (for the most current date, at each nowcast day t the delay distribution is estimated for the complete past) with the empirical delay distribution from the sampled data:

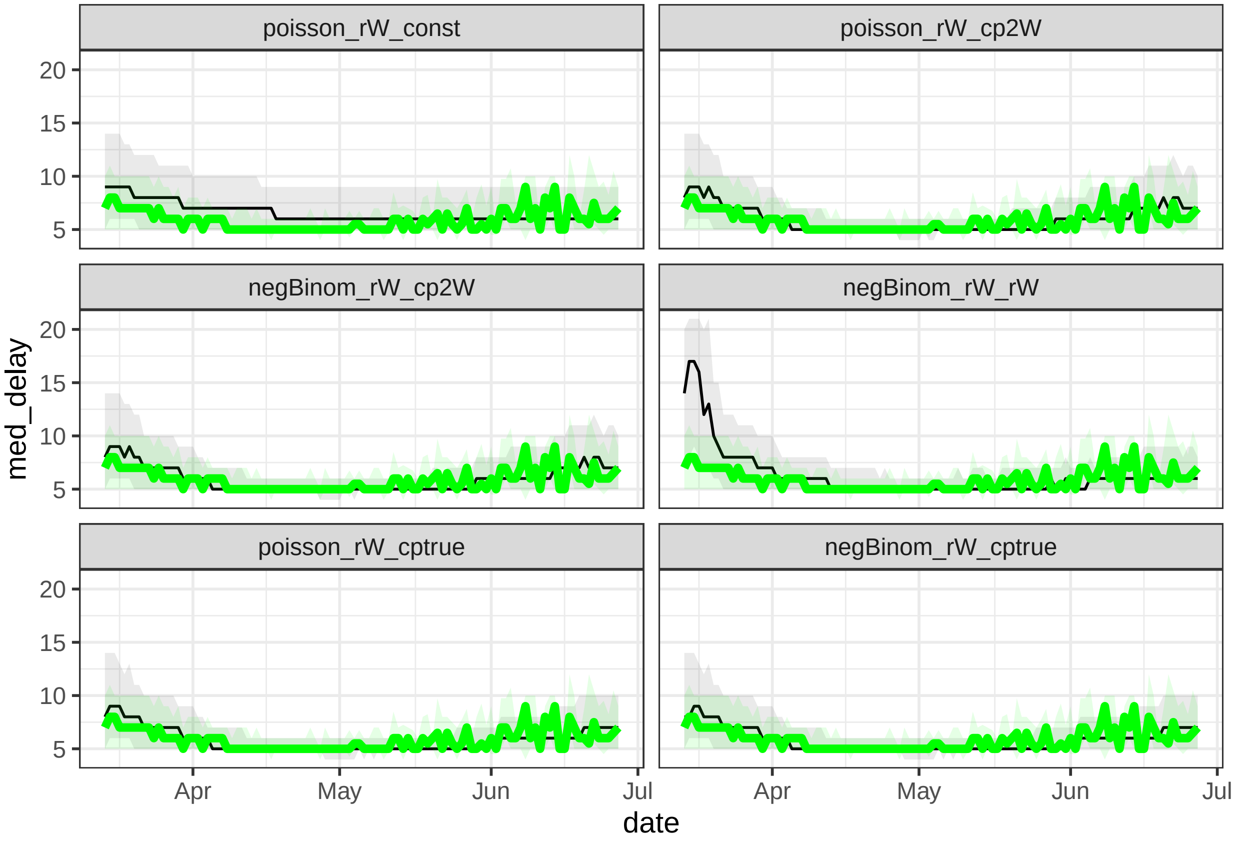

Figure 4: Comparison of estimated delay distribution (black) and empirical delay distribution (blue) per day for each model. Shown are the median and 25% and 75% quantiles.

We summarize the results the following way: when we supply the true, in reality unknown, changepoints of the delay distribution to model fitting, the nowcast performs best with respect to our evaluation metrics. It shows the lowest log and CRPS score, lowest root mean squared error and shows the desired coverage frequencies for the 95%-prediction intervals. With the models assuming changepoints in the linear time effect on reporting delay every two weeks before T, we obtain similar, but slightly worse performance. The approach appears to be able to capture the moderate changes in the delay distribution successfully. Modeling the changes on a daily basis shows a slightly worse performance with respect to the CRPS score and PI coverage frequencies. The prediction intervals are wider and there exists some evidence for convergence problems at the beginning of the nowcast period where to median delay was strongly overestimated leading to an upward bias in the predicted number of newly diseased cases per day. This might be related to the overly complex model for the reporting delay and very few observations to estimate the reporting delay up to this time. Assuming a constant reporting delay distribution over time and ignoring the changes leads to the worst performance with biggest scores and low coverage frequencies of the prediction intervals. The number of estimated newly diseased cases is overestimated during the whole nowcasting period starting at end of March. When specifying an adequate model for the delay distribution, the distributional assumptions regarding $N_{t,d}$ play a minor role for performance on the synthetic data.

Retrospective evaluation based on Bavarian data

For the retrospective evaluation of nowcasting we utilize all official data that was reported until July, 31 and restrict the evaluation period until June, 30, assuming that for all days before, the true number of new cases with disease onset are reported based on the available data at end of July. Furthermore, we focus on all reported cases with available disease onset information. The aggregated case counts in this period are:

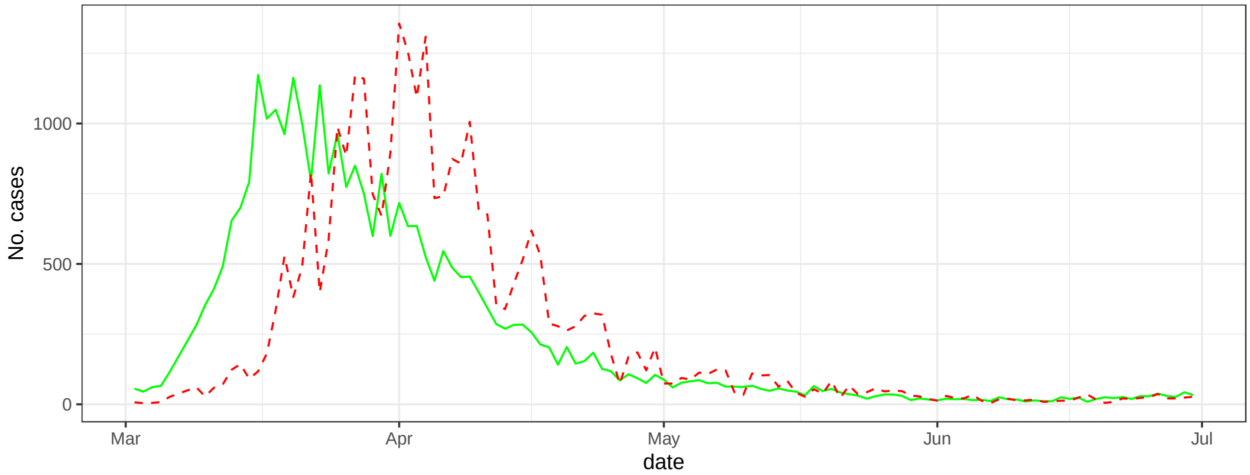

Figure 5: Daily numbers of newly diseased cases and reported cases in Bavarian data. Numbers of disease onsets are derived retrospectively based on data available on July, 31.

The empirical reporting delay distribution can be illustrated as in case of the synthetic data above:

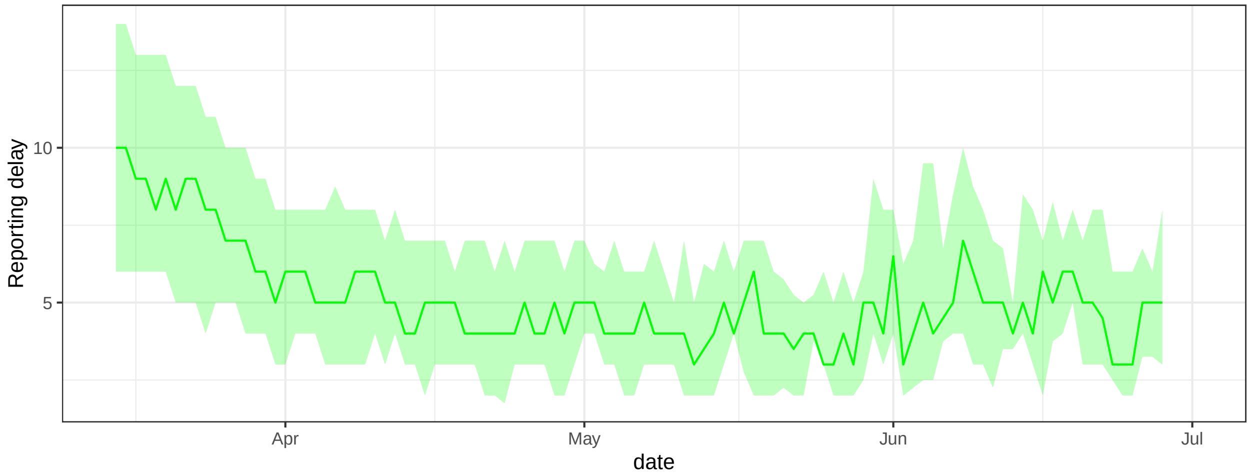

Figure 6: Empirical reporting delay distribution (between disease onset and reporting at LGL) for the Bavarian COVID-19 data.

Note that the illustrated delay is, in contrast to the delay reported in Table 2, the time between disease onset and reporting at LGL (regional health authority). In Table 2, we described the delay between disease onset and reporting at the local health authority that is relevant for the disease onset imputation model. ### Results

We esimated nowcasts for all dates t from March, 17 (22 days after disease onset of first case) until June, 30 by restricting the data to all cases reported until the respective date and compared the nowcast predicitions for all days $t-5, \ldots, t-2$ to the numbers of newly diseased cases per day reported until July, 31. Nowcasts are performed based on six different models (see description in the Paper), and the performance of the models

is compared as above. In addition we compute the coverage of the 95%-credibility intervals of the estimated time-varying reproduction number with the estimate obtained from utilizing all available data unti July, 31.

Table 2: Retrospective quantification of the performance of six different nowcast models on Bavarian COVID-19 data. Shown are the average metrics over all nowcast dates (*_f) and restricted to the period until May, 1 (*_m). Reported scores are the continuous ranked probability score, logarithmic score, root mean squared error of posterior median, and coverage frequencies of 95% prediction intervals, as well as coverage frequencies of the estimated R(t) at the most current date. Estimated models are Poisson and Negative Binomial models with 1) (assumed) constant delay distribution, 2) linear time-effects with changepoints every two weeks before now (*_cp2W), 3) daily changes in delay distribution based on a first-order random walk prior (*_rW) and 2) and 3) with additional effects of the weekday of case reporting (*_wd).

model	crps_f	crps_m	logS_f	logS_m	rmse_f	rmse_m	cov_f	cov_m	cov_Rt
poisson_rW_const	193.43	385.62	Inf	Inf	389.56	570.81	0.19	0.09	0.56
poisson_rW_cp2W	74.32	154.11	Inf	Inf	226.90	333.46	0.67	0.45	0.86
negBinom_rW_cp2W	61.79	127.70	5.05	6.92	205.59	302.15	0.84	0.74	0.92
negBinom_rW_rW	79.21	165.71	4.83	6.53	274.70	403.87	0.86	0.78	0.95
negBinom_rW_cp2W_wd	56.63	117.21	5.22	7.36	170.59	250.70	0.82	0.69	0.92
negBinom_rW_rW_wd	67.32	140.47	4.99	6.88	236.05	347.05	0.90	0.84	0.94

The comparison of the daily nowcast predictions 3 day before now and the retrospective truth, as well as the estimated delay distribution are shown in the following figures:

We find that the Poisson model assuming no changes in the reporting delay distribution performs bad. Daily case numbers are strongly overestimated. This is in line with the apparent changes in the reporting delay between disease onset and reporting at LGL over time. Comparing the Poisson model with two-week changepoints with a similar model using a Negative Binomial distribution for $N_{t,d}$ we find the latter to perform better with respect to the evaluation metrics. Furthermore, it is apparent that prediction intervals in the poisson model are too narrow. This improves when using a Negative Binomial model with overdispersion. Adding weekday effects to the delay distribution improves the performance of the models as well. Comparing the Negative Binomial model with daily changes in the delay distribution with the two week changepoint model, we found better coverage frequencies for the former (mainly because of wider prediction intervals) but lower CRPS score and RMSE for the latter. The reporting delay estimation allowing for daily changes based on a first-order random walk appeared to be somewhat instable at the beginning of the nowcasting period, as also seen in the evaluation based on synthetic data.

Looking visually at the predictions of the nowcast, we find that the predictions close to now (e.g. 3 days before now), as illustrated in the Fig. 7, overestimate daily case counts in the crucial timeperiod between March 15 and April 1. This is also true for our preferred model, with 2 week changepoints and weekday effects in the delay distribution. In this period the case counts of newly diseased individuals stabilized, but daily reported cases were still increasing steadily. The situation of a stabilizing number of new disease onsets and simultaneously decreasing average reporting delay between disease onset and case registration (that becomes now - in retrospect- apparent) is not easily identifiable based on incomplete data in real time surveillance. It is, however, apparent that the nowcasting approach is valuable in understanding the dynamics of the pandemic better compared to the daily counts of newly reported cases. It helps to illustrate uncertainty with respect to the current state of the pandemic and the predictions of our preferred models that account for changes in reporting delay are not too far off. Furthermore coverage frequencies of 95% prediction intervals close to 90% appear acceptable. Looking more closely at the results of e.g., the Negative Binomial model with 2-week changepoints and weekday effects in the delay distribution, we find that based on the nowcast,

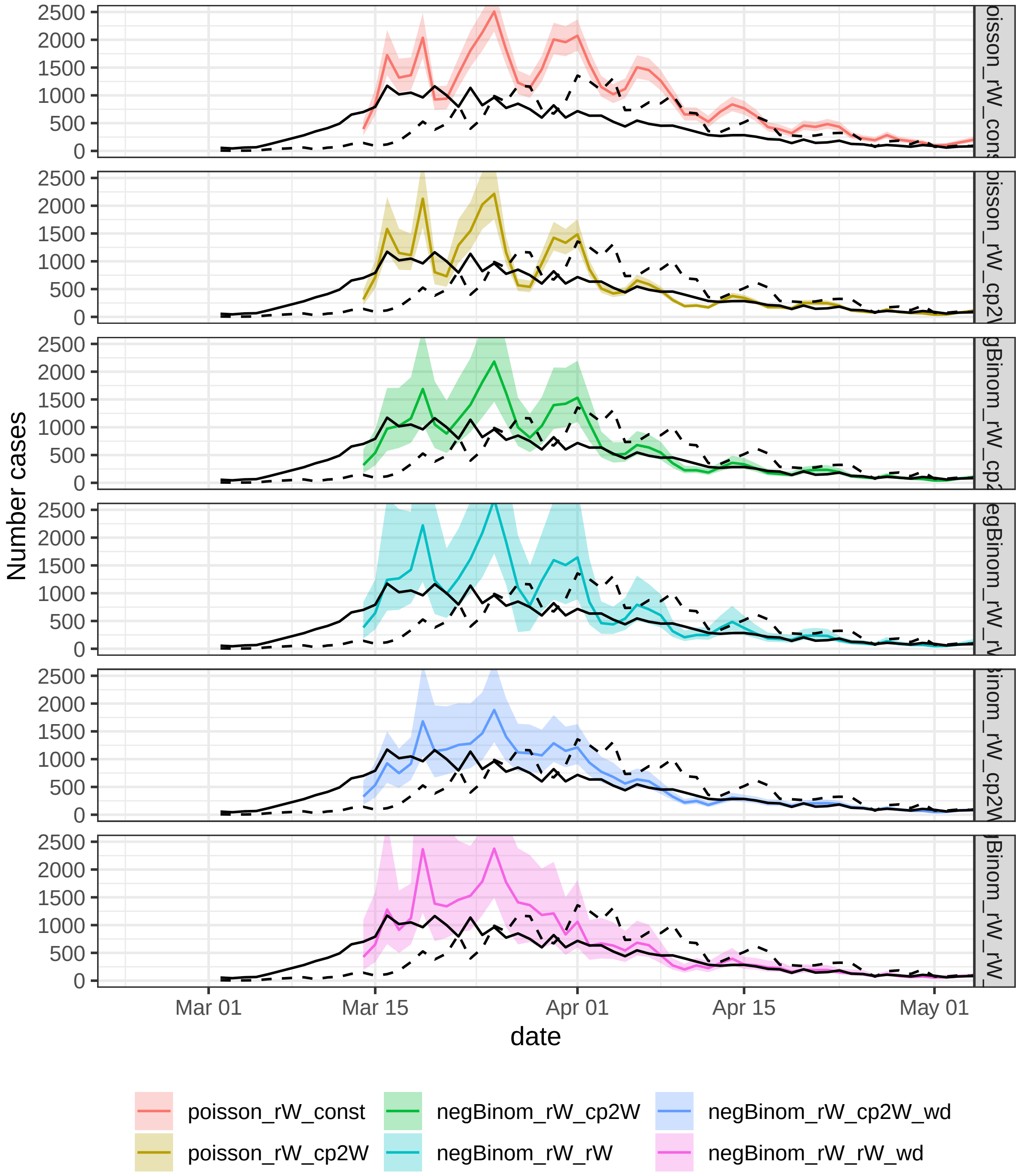

Figure 7: Nowcast predictions and 95% PIs for nowcasts 3 days before 'now' compared to retrospectively true number of disease onsets (solid) and number of reported cases (dotted) in Bavarian data.

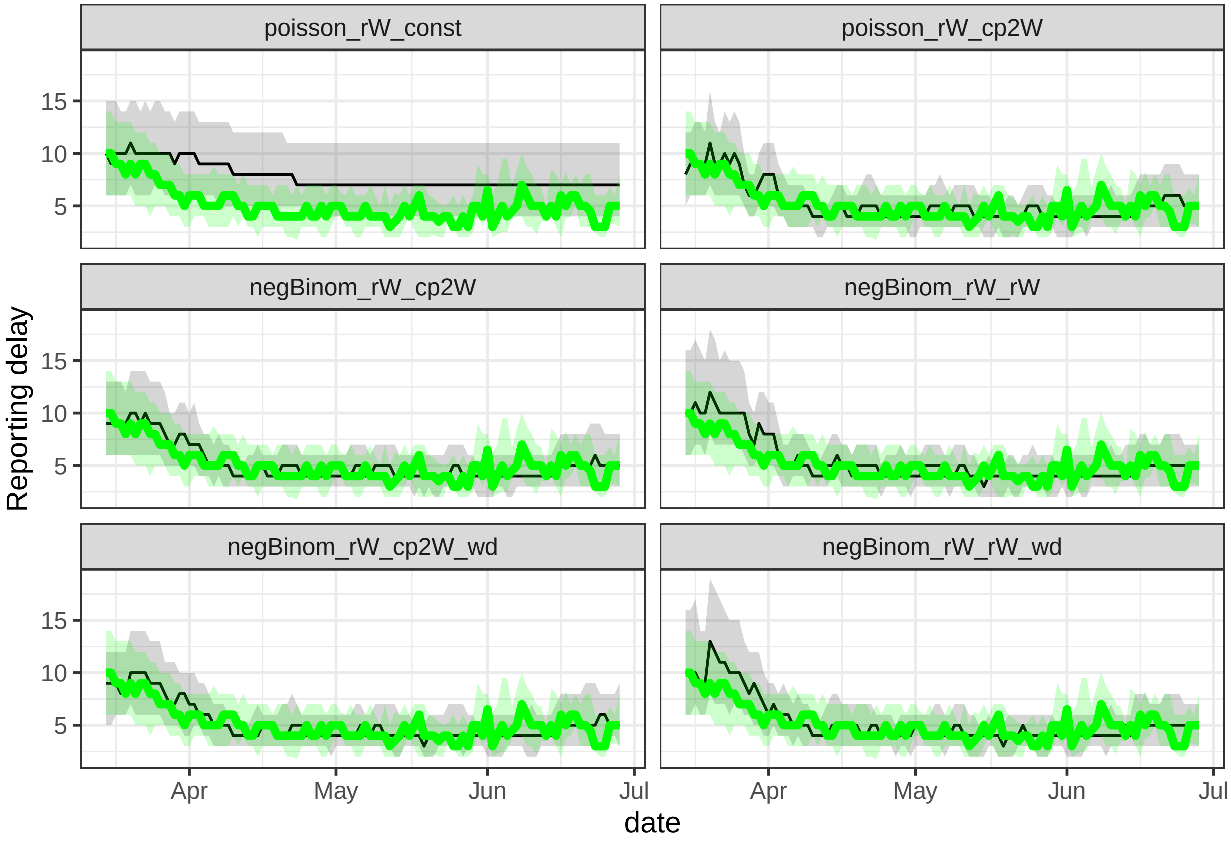

Figure 8: Comparison of estimated delay distribution (black) and retrospective empirical delay distribution (blue) per day for each model on Bavarian data. Shown are the median and 25% and 75% quantiles.

the pandemic situation seems to stabilize from around March, 20 on, and the predictions close to now are already starting to decreas at around April, 1, where the daily number of newly reported cases were still at their peak.

Comparing the estimated $R(t)$ at most current t based on the different nowcast models with the retrospective *truth* based on all reported data, we find coverage probabilities of the 95%− credibility intervals bigger than 90% for all models that consider changes in the delay distribution over time. The estimation of $R(t)$ is, however, biased when it is based on a biased nowcasting approach, e.g., due to ignoring changes in the delay distribution. In the Poisson model assuming no changes in delay distribution $R(t)$ biased upwards during the whole timeperiod.

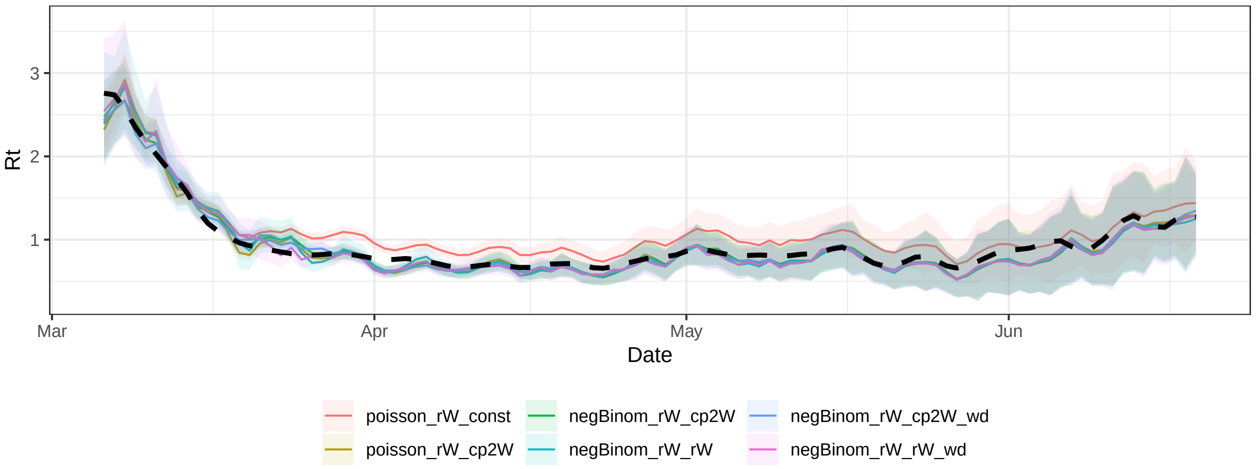

Figure 9: Estimated R(t) over time based on all nowcast models at most current date and associated 95% CI. Comparison with R(t) based on all reported disease onsets until July, 31.
