# Supplementary figures and images for: Nowcasting the COVID‐19 pandemic in Bavaria
Source: Biom J. 2020 Dec 1;63(3):490–502. doi: 10.1002/bimj.202000112 (PMC7753318; doi:10.1002/bimj.202000112)

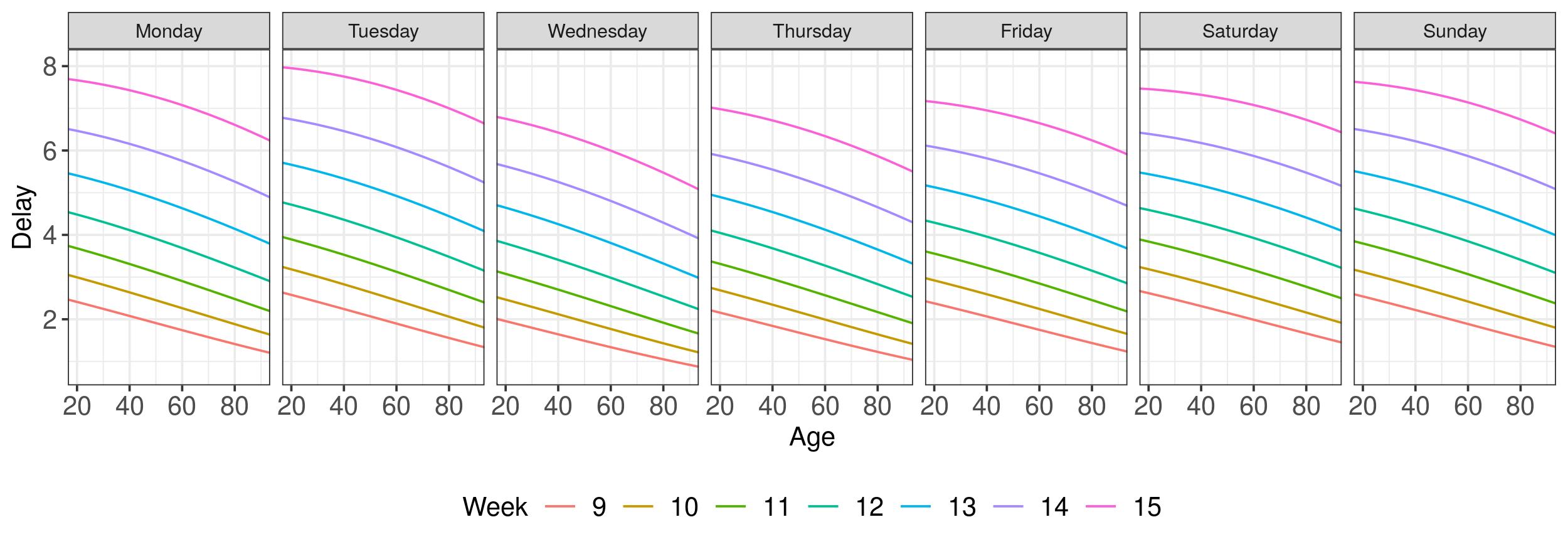

Supplement: Supplementary file 2 — Supporting Information [file BIMJ-63-490-s001.zip › code_and_data/results_public/1_main_analysis/figures/fig_1_imp_synth.jpg]

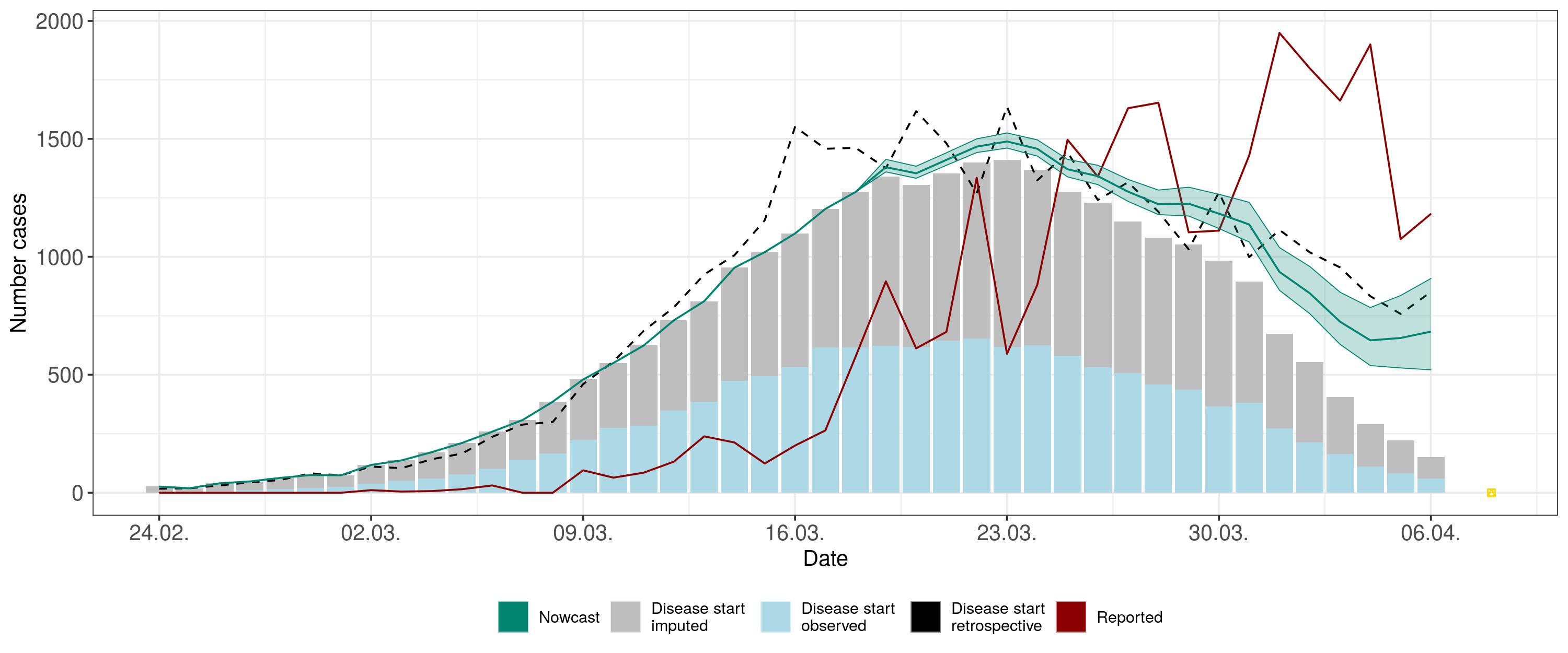

Supplement: Supplementary file 2 — Supporting Information [file BIMJ-63-490-s001.zip › code_and_data/results_public/1_main_analysis/figures/fig_2_nc_synth.jpg]

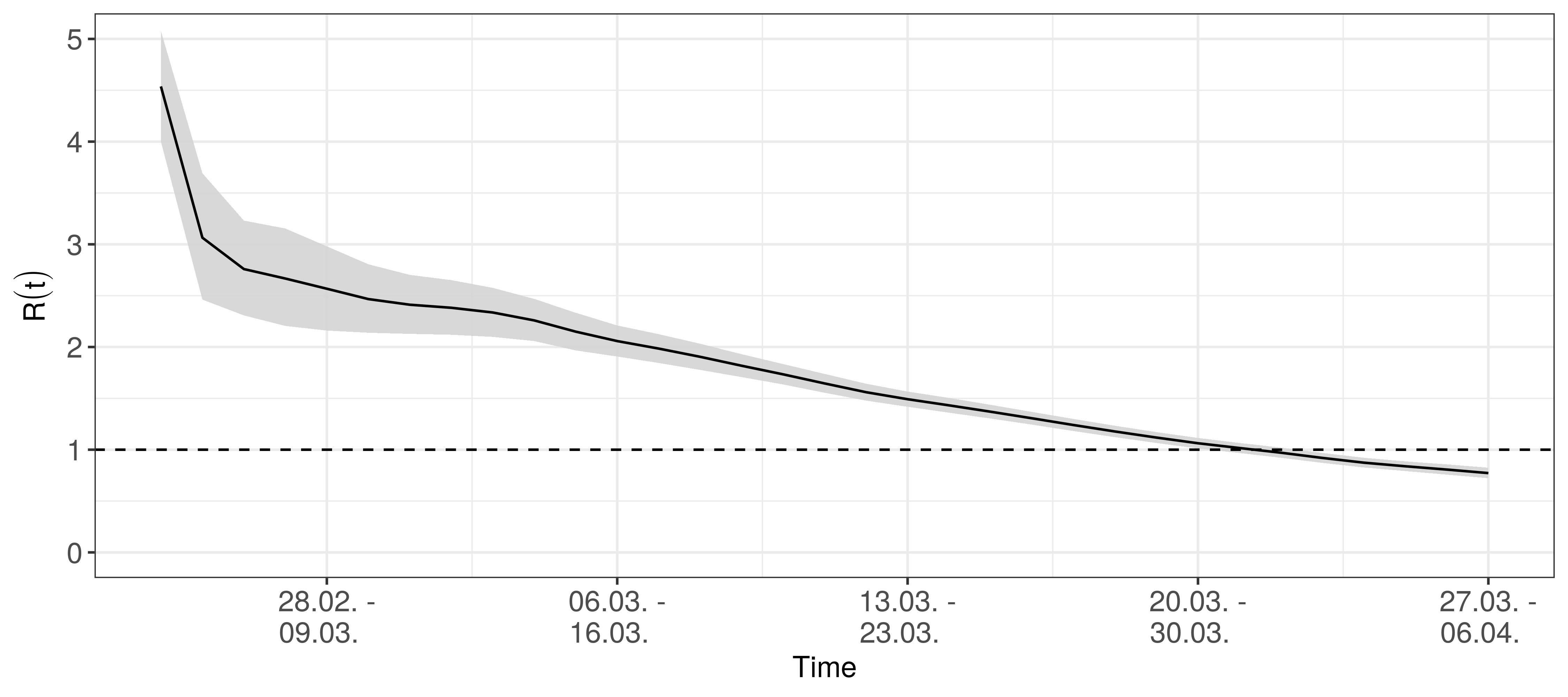

Supplement: Supplementary file 2 — Supporting Information [file BIMJ-63-490-s001.zip › code_and_data/results_public/1_main_analysis/figures/fig_3_Rt_synth.jpg]

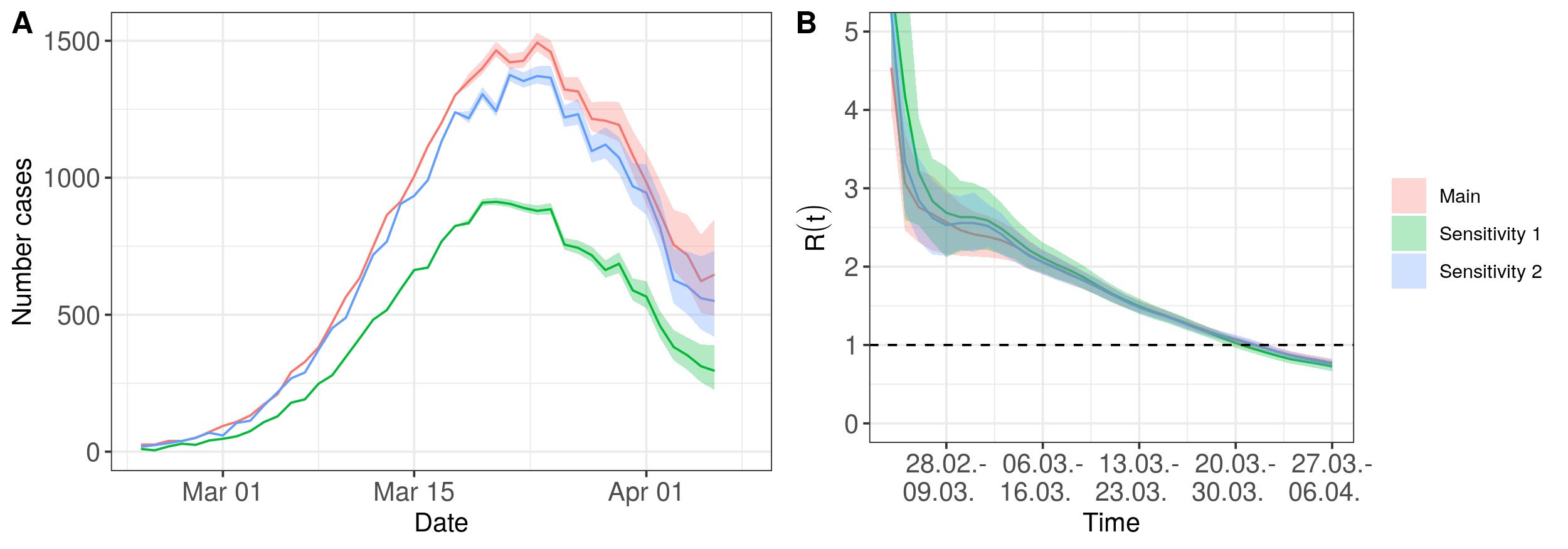

Supplement: Supplementary file 2 — Supporting Information [file BIMJ-63-490-s001.zip › code_and_data/results_public/1_main_analysis/figures/fig_4_sens_synth.jpg]

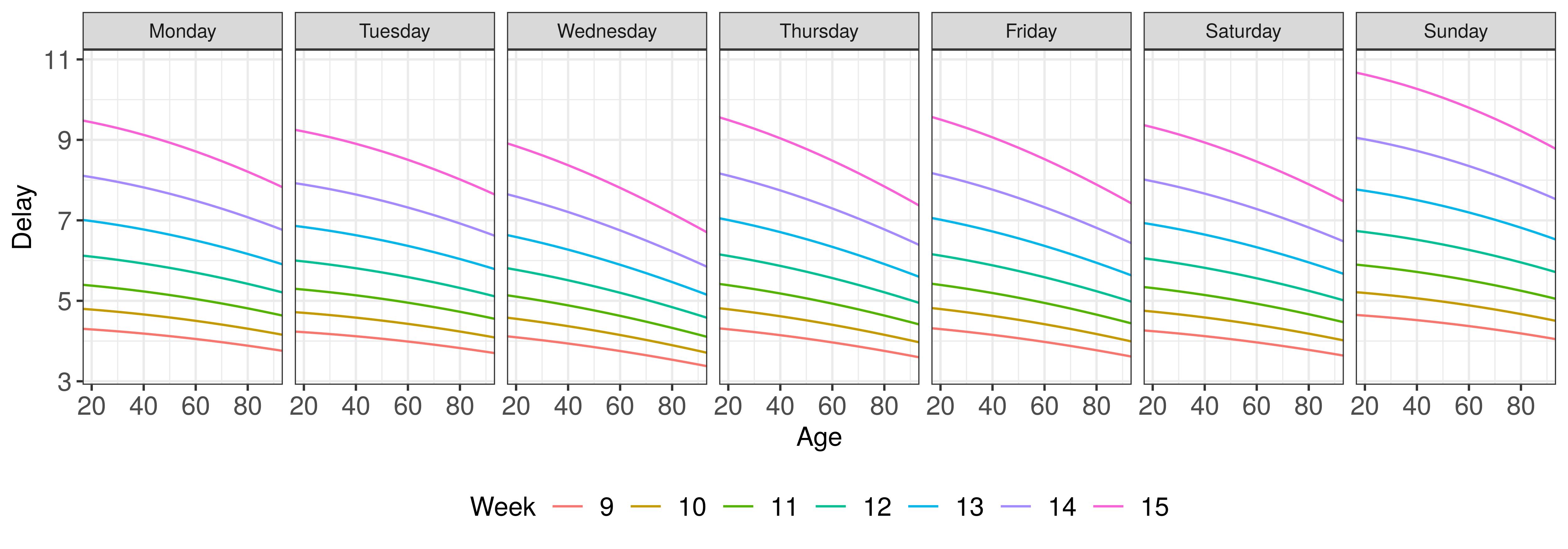

Supplement: Supplementary file 2 — Supporting Information [file BIMJ-63-490-s001.zip › code_and_data/results_public/1_main_analysis/figures/fig_1_imp.jpg]

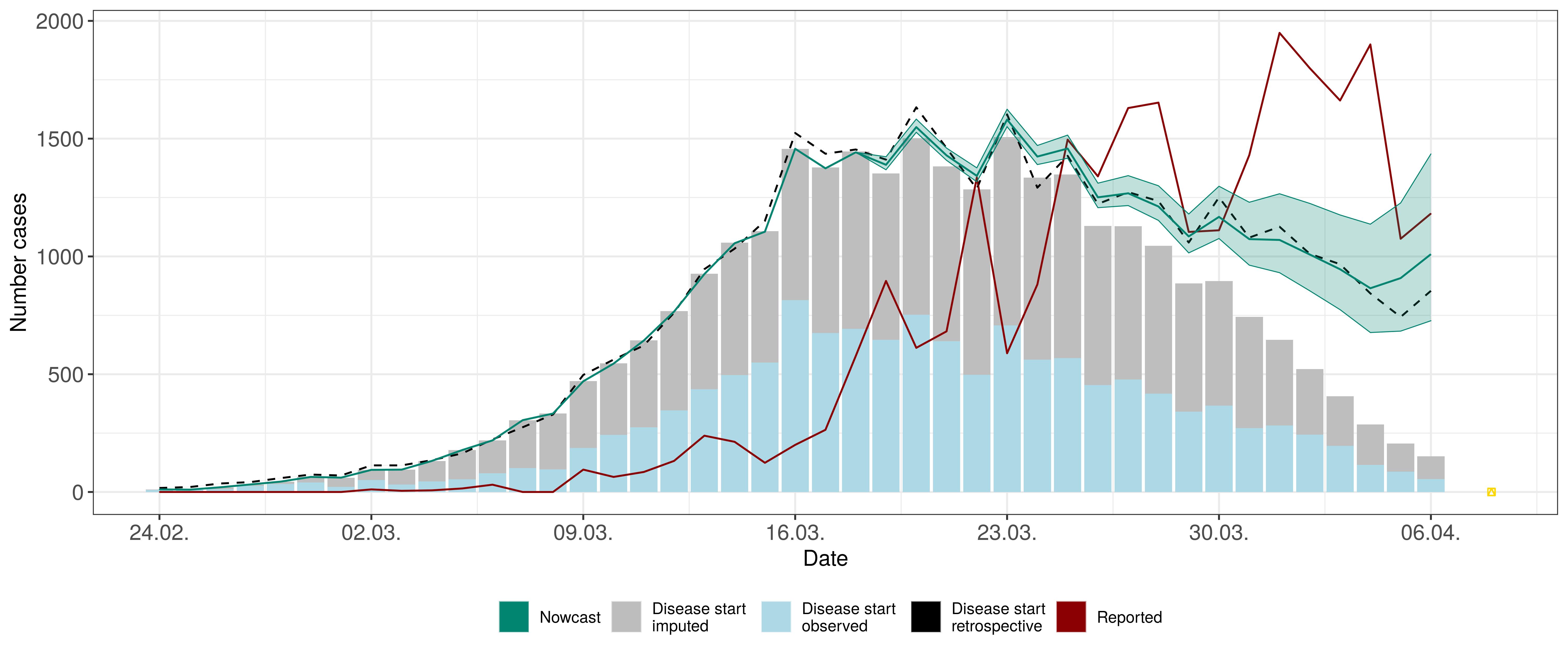

Supplement: Supplementary file 2 — Supporting Information [file BIMJ-63-490-s001.zip › code_and_data/results_public/1_main_analysis/figures/fig_2_nc.jpg]

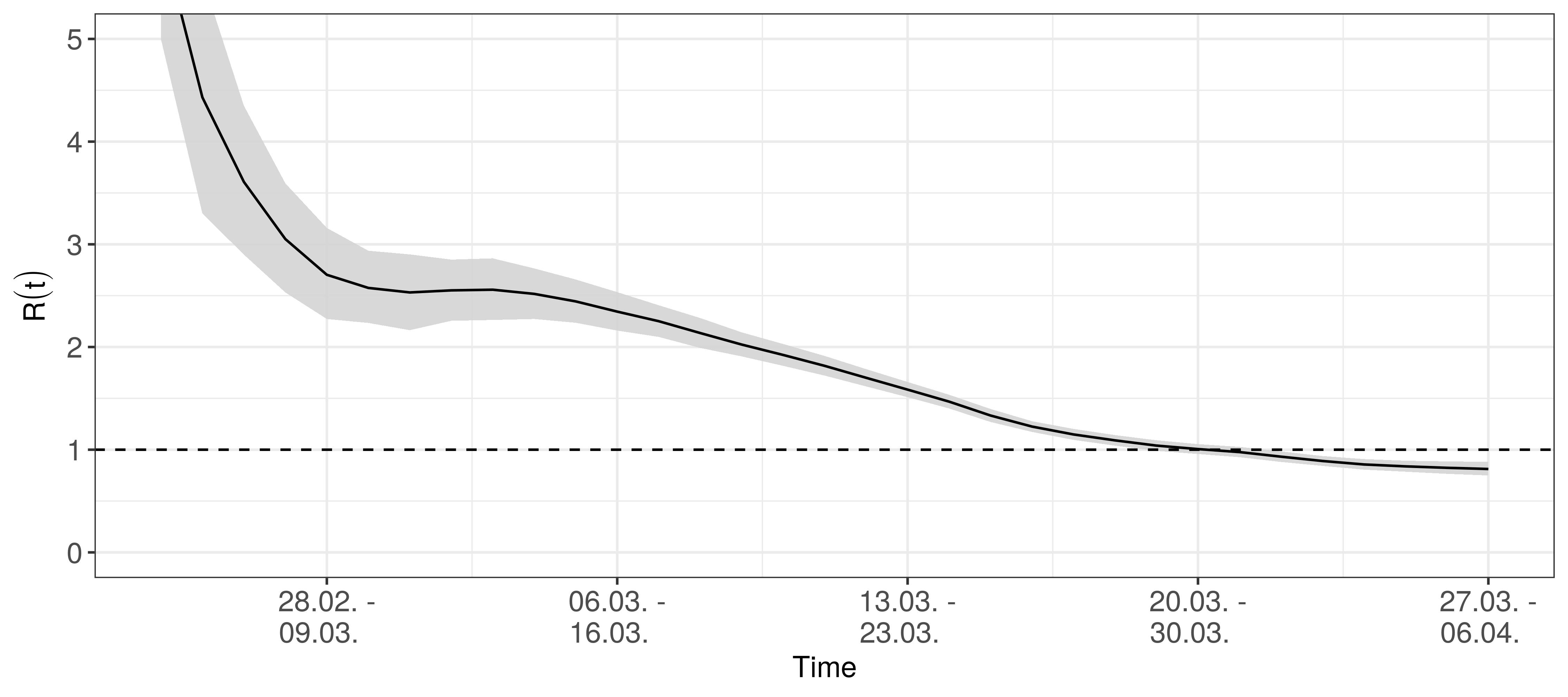

Supplement: Supplementary file 2 — Supporting Information [file BIMJ-63-490-s001.zip › code_and_data/results_public/1_main_analysis/figures/fig_3_Rt.jpg]

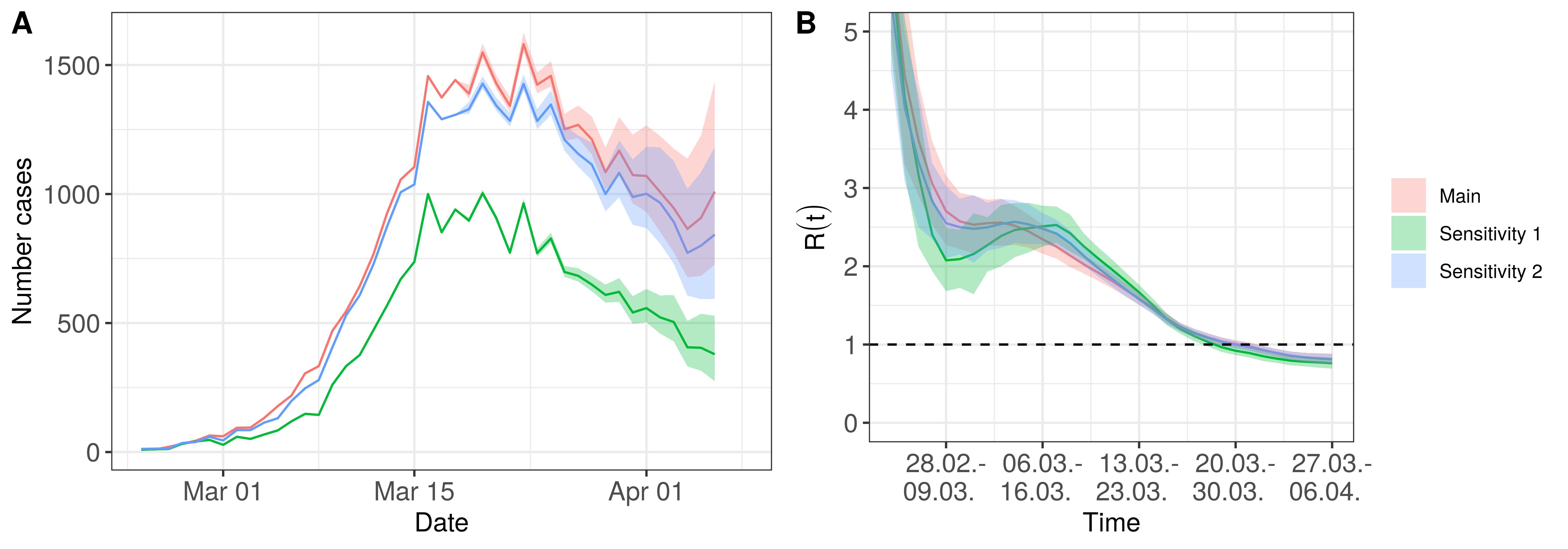

Supplement: Supplementary file 2 — Supporting Information [file BIMJ-63-490-s001.zip › code_and_data/results_public/1_main_analysis/figures/fig_4_sens.jpg]
